# Early goal-directed therapy in severe sepsis and septic shock: insights and comparisons to ProCESS, ProMISe, and ARISE

**DOI:** 10.1186/s13054-016-1288-3

**Published:** 2016-07-01

**Authors:** H. Bryant Nguyen, Anja Kathrin Jaehne, Namita Jayaprakash, Matthew W. Semler, Sara Hegab, Angel Coz Yataco, Geneva Tatem, Dhafer Salem, Steven Moore, Kamran Boka, Jasreen Kaur Gill, Jayna Gardner-Gray, Jacqueline Pflaum, Juan Pablo Domecq, Gina Hurst, Justin B. Belsky, Raymond Fowkes, Ronald B. Elkin, Steven Q. Simpson, Jay L. Falk, Daniel J. Singer, Emanuel P. Rivers

**Affiliations:** Department of Medicine, Pulmonary and Critical Care Medicine, Loma Linda University, Loma Linda, CA USA; Department of Emergency Medicine, Loma Linda University, Loma Linda, CA USA; Department of Emergency Medicine, Henry Ford Hospital, Wayne State University, Detroit, MI USA; Division of Pulmonary and Critical Care Medicine, Mayo Clinic Rochester, Rochester, MN USA; Department of Medicine, Pulmonary and Critical Care Medicine, Vanderbilt University, Nashville, TN USA; Department of Medicine, Pulmonary and Critical Care Medicine, Henry Ford Hospital, Wayne State University, Detroit, MI USA; Department of Medicine, Pulmonary and Critical Care Medicine, University of Kentucky, Lexington, KY USA; Department of Internal Medicine, Mercy Hospital Medical Center, Chicago, IL USA; Department of Internal Medicine, Division of Critical Care Medicine, University of Texas Health Science Center at Houston, Houston, TX USA; Department of Internal Medicine, Henry Ford Hospital, Wayne State University, Detroit, MI USA; CONEVID, Conocimiento y Evidencia Research Unit, Universidad Peruana Cayetano Heredia, Lima, PERU; Department of Emergency Medicine, Massachusetts General Hospital, Harvard Medical School, Boston, MA USA; Pulmonary and Critical Care Medicine, California Pacific Medical Center, San Francisco, CA USA; Pulmonary and Critical Care Medicine, University of Kansas, Kansas City, Kansas USA; Department of Emergency Medicine, Orlando Regional Medical Center, Orlando, Florida USA; University of Central Florida College of Medicine, Orlando, Florida USA; University of Florida College of Medicine, Orlando, Florida USA; University of South Florida College of Medicine, Orlando, Florida USA; Florida State University College of Medicine, Orlando, Florida USA; Department of Surgery, Division of Surgical Critical Care, Icahn School of Medicine, Mount Sinai Hospital,, New York, NY USA; Department of Surgery, Henry Ford Hospital, Wayne State University, Detroit, MI USA; Department of Quality Assurance, Aspirus Hospital, Iron River, MI USA

## Abstract

Prior to 2001 there was no standard for early management of severe sepsis and septic shock in the emergency department. In the presence of standard or usual care, the prevailing mortality was over 40-50 %. In response, a systems-based approach, similar to that in acute myocardial infarction, stroke and trauma, called early goal-directed therapy was compared to standard care and this clinical trial resulted in a significant mortality reduction. Since the publication of that trial, similar outcome benefits have been reported in over 70 observational and randomized controlled studies comprising over 70,000 patients. As a result, early goal-directed therapy was largely incorporated into the first 6 hours of sepsis management (resuscitation bundle) adopted by the Surviving Sepsis Campaign and disseminated internationally as the standard of care for early sepsis management. Recently a trio of trials (ProCESS, ARISE, and ProMISe), while reporting an all-time low sepsis mortality, question the continued need for all of the elements of early goal-directed therapy or the need for protocolized care for patients with severe and septic shock. A review of the early hemodynamic pathogenesis, historical development, and definition of early goal-directed therapy, comparing trial conduction methodology and the changing landscape of sepsis mortality, are essential for an appropriate interpretation of these trials and their conclusions.

**Electronic supplementary material:**

The online version of this article (doi:10.1186/s13054-016-1288-3) contains supplementary material, which is available to authorized users.

## Background

### The early physiologic-hemodynamic response to severe sepsis and septic shock

In animal and human models of early sepsis, global tissue hypoxia results from hemodynamic perturbations that create an imbalance between systemic oxygen delivery and demands. These perturbations can include hypovolemia, decreased vasomotor tone, decreased arterial oxygen content, myocardial depression, increased metabolic demands, and impairment of systemic oxygen utilization via microcirculatory or mitochondrial derangements (cytopathic tissue hypoxia) [1]. A critical decrease in systemic oxygen delivery is followed by an increase in the systemic oxygen extraction ratio and a decrease in mixed or central venous (SvO_2_ or ScvO_2_) oxygen saturation. Anaerobic metabolism ensues when the limits of this compensatory mechanism cannot maintain systemic oxygen consumption leading to lactate production [2]. The final, and often terminal, stage is an impairment of systemic oxygen utilization. Patients in this stage have elevated ScvO_2,_ increased lactate, and decreased systemic oxygen consumption (Additional file 1: Figure S1).

As a result of this early response, distinct hemodynamic phenotypes emerge. Characterizing patients by distinct hemodynamic phenotypes using ScvO_2_, lactate, and blood pressure provides diagnostic, therapeutic, and prognostic staging of sepsis for study comparisons. These hemodynamic phenotypes reflect distinct stages along a continuum of disease whether pre-hospital, in the emergency department (ED), on general practice floors or in the intensive care unit (ICU) setting.

### The history and development of early goal-directed therapy (EGDT)

**Fig. 1:**
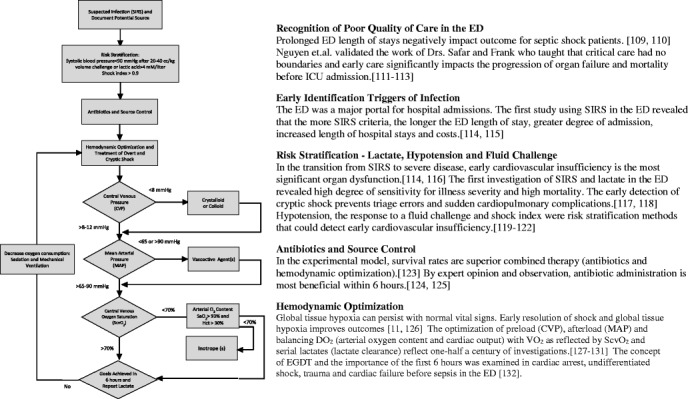
A systems-based approach. The origin and components of EGDT. *Hct* hematocrit [109–132]

Beginning in the early 1990s, the EGDT Collaborative Group challenged the paradigm of sepsis care as an “ICU disease” by applying similar urgent diagnostic and therapeutic principles as used for acute myocardial infarction, stroke, and trauma at the point of presentation in the ED. At that time, no structured formal worldwide accepted protocols for early identification and treatment of patients with sepsis existed. The observations of high mortality, fractured, and unstructured care triggered a series of investigations using a system-based approach to identify delays in patient diagnosis and care before hospital admission. Combining system issues with the early pathogenesis and natural progression of sepsis required the development of unique diagnostic and risk stratification criteria to detect patients at risk and most likely to benefit (Fig. 1) [3].

### From EGDT to ProCESS, ARISE and ProMISe

EGDT is comprised of early identification of high-risk patients, appropriate cultures, source control, and administration of appropriate antibiotics. This is followed by early hemodynamic optimization of oxygen delivery, guided by preload (central venous pressure (CVP) or surrogate targeting with fluids), afterload (mean arterial pressure (MAP) targeted with vasopressors), arterial oxygen content (packed red blood cells and/or oxygen supplementation), contractility (inotropic agents), and decreasing oxygen consumption (mechanical ventilation and sedation), and guided by ScvO_2._ These principles were essentially best practice recommendations for sepsis management in the ICU setting (Fig. 1) [4].

After observing a local hospital mortality of over 50 % for severe sepsis and septic shock, an institutional quality improvement initiative led to the randomized controlled trial of EGDT between 1997 and 2000 [3]. After validity, reliability, and feasibility testing across multiple healthcare settings both nationally and internationally for over a decade, EGDT became part of the fundamental components of the sepsis resuscitation bundle for the Surviving Sepsis Campaign (SSC), the National Quality Forum and Centers for Medicare and Medicaid Services [5].

Since the EGDT publication, significant scientific interest was generated to "disassemble or unbundle" early sepsis resuscitation and question the value of its individual components [6, 7]. Even though EGDT was based on a series of investigations to systematically improve sepsis outcomes, it has been inappropriately characterized as a hemodynamic optimization study driven by CVP and ScvO_2_ as targets for early shock resolution [8–11]. There was also the additional question of its external validity because it was a single center study with an “unusually high” control group mortality of 46.5 %. Recently, a "trio of trials" which examined versions of EGDT called ProCESS (Protocol-Based Care for Early Septic Shock), ARISE (Australasian Resuscitation in Sepsis Evaluation) and ProMISe (Protocolized Management in Sepsis) were published from a related consortium of investigators [12–14].

The trio of trials of EGDT reported an unprecedented all-time low in sepsis mortality for all treatment groups compared to historical controls (Table 1). However, they concluded: "EGDT does not show improved survival for patients randomized to receive EGDT compared to usual care or to less invasive alternative hemodynamic resuscitation protocols. EGDT is, however, associated with increased admission to ICU. Our findings do not support the systematic use of EGDT in the management of all patients with septic shock or its inclusion in the Surviving Sepsis Campaign guidelines” [15]. The purpose of this review is to provide the reader with the critical information needed to objectively interpret the purpose, methodology, results, and conclusion of the trio of EGDT trials [16–19].

**Table 1:** Comparison of observational studies before and during the EGDT, ProCESS, ProMISe and ARISE trials

Studies	Year	Mortality before (%)^a^	Mortality after (%)^b^
EGDT [3]	1997–2000	46.5	30.5
Shanker-Hari et al. (septic shock) [96] (n = 52, n = 166,479)	1993-2015	46.5	n/a
US observational Studies			
Dombrovsky et al. (severe sepsis) [133]	2001	40.3	n/a
Ani et al. (severe sepsis) [134]	1999–2008	40.0	27.8
Stevenson et al. [135]	1993–2009	46.9	29.2
Kumar et al. (severe sepsis) [136]	2003–2009	39.6	27.3
Kumar et al. (septic shock) [136]	2000–2007	47.1	36.4
Mechanically ventilated patients [60]	2002–2012	64.1	39.7
Studies of EGDT (number of studies, number of patients)			
Quasi experimental studies (*n* = 4, *n* = 1120) [137–140]	2001–2016	45.8	28.5
Prospective observational (*n* = 38, *n* = 66,862) [43, 87, 91, 93, 94, 141–174]	2001–2016	40.3	27.6
Prospective with historical controls (*n* = 9, *n* = 2250) [175–183]	2001–2016	45.5	29.6
Retrospective (*n* = 10, *n* = 2183) [184–193]	2001–2016	41.1	24.7
Randomized control trials (*n* = 11, *n* = 5756) [3, 12–14, 79, 194–199]	2001–2016	31.3	26.2
ProCESS [12]	2008–2013	18.9	19-20
United Kingdom observational studies			
Padkin et al. [200]	1995–2000	47.0	n/a
Gao et al. [148]	2004–2005	55.0	29.0
Reuben et al. [201]	2004–2005	43.0	n/a
Melville et al. [202]	2005–2008	51.9	41.3
Daniels et al. [203]	2007–2008	44.1	20.0
Sivayoham et al. [189]	2006–2009	42.8	22.7
ProMISe [14]	2011–2014	25.6	24.6
Australia and New Zealand observational studies			
Finfer et al. (severe sepsis) [204]	1999	37.5	n/a
Kaukonen et al. (severe sepsis, with co morbidities) [205]	2000–2012	46.3	23.4
Kaukonen et al. (severe sepsis) [205]	2000–2012	30.2	14.2
Kaukonen et al. (septic shock) [205]	2000–2012	40.3	22.0
ARISE [13]	2008–2014	18.8	18.6

^a^Before (baseline, usual or control); ^b^After (treatment). References are given in Additional file 1 (Table S6)

*ARISE* Australasian Resuscitation in Sepsis Evaluation, *EGDT* Early Goal-Directed Therapy, *ProCESS* Protocolized Care for Early Septic Shock, *ProMISe* Protocolized Management in Sepsis

## Review

### Enrollment procedures and logistics

The location, number of centers, hospital setting and size, and number of ED visits for the EGDT and trio of EGDT trials are noted in Table 2 and Additional file 1, Table S1. Trials were primarily conducted in academic/tertiary care centers, where higher patient volumes are associated with better outcomes [20]. The trio of EGDT trials began 8 years after completion of the EGDT trial, were conducted over a 5-year period, and published more than 14 years after the EGDT trial. This time period also paralleled the introduction (2004) and two revisions of the SSC guidelines in 2008 and 2013 [5].

**Table 2:** Enrollment characteristics and data

	EGDT		ProCESS			ARISE		ProMISe	
Treatment groups	EGDT	Control	EGDT	PBST	Usual	EGDT	Control	EGDT	Usual
Location	United States		United States			Multinational^a^		United Kingdom	
Number of centers	1		31			51^a^		56	
Setting	Metropolitan academic teaching hospital		Metropolitan academic teaching hospitals			Metropolitan and rural tertiary and non-tertiary care teaching hospitals		National Health Service hospitals throughout the United Kingdom	
Enrollment time frame	March 1997–March 2000		March 2008–May 2013			October 2008–April 2014		February 2011–July 2014	
Duration of study (months)	36		62			66		41	
Patients enrolled	263		1341			1600		1260	
Eligible patients excluded	10.4 %		65.0 %			42.7 %		66.6 %	
Enrollment/month/center	7		0.7			0.5		0.5	
Lactate screening program	For enrollment		Required			Required		Required	
Existing sepsis protocols	No		Yes (SSC and individual center protocols)			Yes (SSC and national standards)		Yes (SSC and national standards)	
Fluid challenge	20–30 mL/kg		Initially, 20 mL/kg; changed to 1000 mL (55 % enrolled using latter criteria)			1000 mL (70 % of patients)		1000 mL	
Location of study	ED		ED/ICU			ED/ICU		ED/ICU	
Blinding of ICU clinicians	Yes		No			No		No	
Treatment team structure	ED attending, resident, nurses (clinical care)		Study physician/attending, study coordinator, nurse			ED or ICU MD consultant, registrar, or nurse		ED or ICU MD consultant, registrar, or nurse	
Hours to randomization	1.3	1.5	3.3	3.1	3.0	2.8	2.7	2.5	2.5
ED length of stay (hours)	8.0	6.3	Not reported			1.4	2.0	1.2	1.2

^a^Number of study sites by country—Australia: 42 sites, New Zealand: 3 sites, Finland: 2 sites, Ireland: 1 site and Hong Kong: 3 sites

*ARISE* Australasian Resuscitation in Sepsis Evaluation, *ED* emergency department, *EGDT* Early Goal-Directed Therapy, *ICU* intensive care unit, *MD* Medical Doctor, *PBST* protocol-based standard therapy, *ProCESS* Protocolized Care for Early Septic Shock, *ProMISe* Protocolized Management in Sepsis, *SSC* Surviving Sepsis Campaign

Eligible patients were excluded in 10.4 %, 65 %, 42.7 %, and 55.4 % of the EGDT, ProCESS, ARISE, and ProMISe trials, respectively. The enrollment rate was 7 patients per month for the EGDT trial compared with 0.5 to 0.7 patients per month per center in the trio of EGDT trials (Table 2). Daytime and weekday enrollment (as in the ProMISe trial) is associated with lower mortality when compared to nighttime and weekends [21, 22]. High exclusion rates, convenient enrollment, low patient per site relative to high-volume recruitment, and a 5-year duration of enrollment methodologically challenges the external validity of even large randomized trials [19].

### Baseline enrollment criteria (SIRS, lactate, and blood pressure)

An increased respiratory rate, lower partial pressure of carbon dioxide, and decreased temperature were the more prominent systemic inflammatory response syndrome (SIRS) criteria in the EGDT study patients. Additionally, the EGDT study patients had a greater degree of metabolic acidosis and lower ScvO_2_, reflecting greater shock severity (Table 3) [23–25]. While lactate remains an excellent early screening tool, the incidence of a normal lactate level in septic shock is frequent, necessitating an alternative method of risk stratification such as hypotension [26, 27]. On the other hand, intermediate lactate levels (2–4 mM/L) are also associated with increased mortality which is significantly reduced (19 % odds ratio for hospital mortality) with protocolized care [28–32].

**Table 3:** Comparison of enrollment criteria and resuscitation endpoints

	EGDT		ProCESS			ARISE		ProMISe	
	EGDT	Control	EGDT	PBST	UC	EGDT	Control	EGDT	Control
Temperature, °C	35.9	36.6	37.6	37.6	37.7	37.6	37.6		
Heart rate, beats/min	117	114	113.7	114.6	114.5	104.9	104.7		
Systolic blood pressure, mm Hg	106	109	100.2	102.1	99.9	78.8	79.6	77.7	78.4
Respiratory rate, breaths/min	31.8	30.2	25.4	25.1	25.3	24.5	25.1		
Lactate, mM/L	7.7	6.9	4.8	5.0	4.8	4.4	4.2		5.1
Lactate >4, mM/L (%)	79		59	59.2	60.7	46	46.5	65.4	63.7
CVP, mmHg	5.3	6.1				>10			
ScvO_2_, %	48.6	49.2	71			72.7		70.1	
pH	7.31	7.32	7.33	7.31	7.34				
PaCO_2_, mm Hg	31.5	30.6	35.7	38.9	36.9	35.2	35.5		
MAP (6 h), mm Hg	95	81	77	79	76	76.5	75.3	76.5	76.5
CVP (6 h), mmHg	13.8	11.8				11.4	11.9	11.2	11.7
ScvO_2_ (6 h), %	77.3	66.0				75.9		74.2	

Open spaces indicate data not available

*ARISE* Australasian Resuscitation in Sepsis Evaluation, *CVP* central venous pressure, *EGDT* Early Goal-Directed Therapy, *MAP* mean arterial pressure, *PaCO*_*2*_ partial pressure of carbon dioxide, *PBST* protocol-based standard therapy, *ProCESS* Protocolized Care for Early Septic Shock, *ProMISe* Protocolized Management in Sepsis, *ScvO*_*2*_ central venous oxygen saturation, *UC* usual care

A hypotensive episode is associated with an increased risk of death and the response to an adequate fluid challenge improves upon this discriminatory value [32, 33]. The fluid challenge requirement of EGDT (20–30 mL/kg) after randomization into the study was significantly higher than the 1 liter fixed bolus over 60 min prior to randomization used in the trio of EGDT trials (Table 4, Additional file 1: Figure S3 ). Patients enrolled in these trials because they remained hypotensive after 1 liter of crystalloid may not be similarly enrolled in the EGDT trial if they were given 20–30 mL/kg of fluids (Tables 4 and 5).

**Table 4:** Comparison of treatments across the EGDT, ProCESS, ARISE, and ProMISe trials

	EGDT		ProCESS			ARISE		ProMISe	
	EGDT	Control	EGDT	PBST	UC	EGDT	UC	EGDT	UC
Fluid from ED arrival to 6 h, mL^a^	4981	3499	5059	5511	4362	4479	4304	4216	3987
Fluids 6–72 h, mL	8625	10,602	4458	4918	4354	4274	4382	4215	4366
Total fluids 0–72 h, mL	13,443	13,358	7253	8193	6663	6906	6672	5946	5844
Vasopressor 0–6 h, %	27.4	30.3	54.9	52.2	44.1	66.6	57.8	53.3	46.6
Vasopressor 6–72 h, %	29.1	42.9	47.6	46.6	43.2	58.8	51.5	57.9	52.6
Vasopressor 0–72 h, %	36.8	51.3	60.4	61.2	53.7			60.5	55.0
Inotrope 0–6 h, %	13.7	0.8	8.0	1.1	0.9	15.4	2.6	18.1	3.8
Inotrope 6–72 h, %	14.5	8.4	4.3	2.0	2.2	9.5	5.0	17.7	6.5
Mechanical ventilation 0–6 h, %	53.0	53.8	26.4	24.7	21.7	34.8^c^	32.9^c^	20.2	19.0
Mechanical ventilation 6–72 h, %	2.6	16.8	33.7	31.4	27.9	38.6^c^	40.6^c^	24.4	25.4
Any mechanical ventilation, %	55.6	70.6	36.2	34.1	29.6	30.0	31.5	27.4	28.5
Steroids pre-randomization, %	None	None	9.3	9.4	8.3			5	4
Steroids 0–6 h, %	None	None	12.3	10.8	8.1			11.7	11.5
Any steroids 72 h, %	None	None				36.9	35.9	21.9	21.1

^a^The Pre-Randomization period refers to a time-frame prior to the time of informed consent for study enrollment. Interventions were initiated as indicated, but these interventions were not considered for outcome evaluations (Additional file 1: Figure S3)

^b^Combined invasive and non-invasive mechanical ventilation

*ARISE* Australasian Resuscitation in Sepsis Evaluation, *EGDT* Early Goal-Directed Therapy, *ED* emergency department, *PBST* protocol-based standard therapy, *ProCESS* Protocolized Care for Early Septic Shock, *ProMISe* Protocolized Management in Sepsis, *UC* usual care

**Table 5:** Summary of Methodological Comparisons

	The trio of EGDT trials	EGDT study
Requisite for enrollment and defined as usual care	Screening using SIRS Fluid challenge Lactate screening for cryptic shock Early antibiotic administration within 6 h encouraged (ProCESS)	No previous standards. Developed from a series of studies over a decade.
Enrollment	Enrollment (8/site/year) 2- to 12-h window of enrollment in the ED Weekdays and no weekends (ProMISe) Exclusion rate of 43 to 67 %	Single center 1–2 h enrollment
Fluid challenge	Fluid challenge—1 liter or surrogate	20–30 mL/kg
Trial duration and timing	Trials began 7–8 years after EGDT (2008–2015) Duration ranging between 4 and 8 years SSC guidelines were published in 2004, 2008, and 2012	No existing sepsis protocols
Blinding	Open label study in the ICU	ICU was blinded to care provided in the ED
Trial conduct	Duration of ED stay less than 3 h Majority of care provided in ICU Delayed resuscitation bundle completion after 6 h not tested High volume and tertiary care centers CVP placement over 50 % of control groups in trio of EGDT trials A reduction in sample size after interim analysis low mortality	Performed in ED only 6–8 h in the ED Delayed care improves outcomes
Co-morbidities	Fewer Younger patients	Increased cardiovascular, liver, neurologic and renal failure
Mechanical ventilation	Rate of 26 % No delayed increase after enrollment Protective lung strategies	Rate of 54 % No protective lung or fluid management strategies Increase in delayed MV in the control group.
Illness severity	Acute pulmonary edema excluded Acute lung injury excluded	Lower temperature Lower PaCO_2_ More tachypnea
Hemodynamic phenotype	Normal ScvO_2_ and CVP at baseline (all groups received similar fluids as the original EGDT treatment group from hospital arrival to 6 hours) 50 % more vasopressors (vasodilatory) in the trio of EGDT trials Steroid use 8–37 %	Lower ScvO_2_ Higher lactate Lower CVP No steroid use
Sudden cardiopulmonary events	Not a predominant feature	Significant reduction from 20 to 10 %
Sources of improved care	Pre-existing sepsis protocols, pre-hospital care, sepsis alerts and screens, rapid response systems, telemedicine, glucose control, ventilator strategies, hemoglobin strategies, palliative care, national limits on ED length of stay (Australia and United Kingdom), ultrasound	
Generalizability and external validity	Performed in academic centers in industrialized countries Specialized care delivery	EGDT replicated in community and academic centers worldwide

*CVP* central venous pressure, *EGDT* Early Goal-Directed Therapy, *ED* emergency department, *ICU* intensive care unit, *MV* mechanical ventilation, *PaCO*_*2*_ partial pressure of carbon dioxide, *ProCESS* Protocolized Care for Early Septic Shock, *ProMISe* Protocolized Management in Sepsis, *ScvO*_*2*_ central venous oxygen saturation, *SIRS* systemic inflammatory response syndrome, *SSC* Surviving Sepsis Campaign

### Methodology—ED presentation, duration of stay and blinding of care

Randomization and protocol completion was exclusively performed in the ED (minimum of 7–8 h) in the EGDT trial to reflect the reality of care and maximize external validity [28]. This compares to a length of stay of less than 3 h in the ED and the remainder in the inpatient setting in the trio of EGDT trials (Table 2). National initiatives to admit patients to the hospital within 4 h of ED presentation (ProMISe and ARISE) may have improved sepsis care [34, 35]. It has also been noted in the USA that early ICU admission not only improves the processes of care but contributes to diminishing mortality [36, 37].

In the EGDT trial, the admitting inpatient clinicians (ICU) were completely blinded to the randomized treatment group in the ED and clinical variables related to the study during the 72-h follow-up [3, 18]. This included blinding to lactate (and lactate clearance) as well as ScvO_2_ values over 72 h as they were not a standard of care and not readily available in the chart. In contrast, the care provided in the trio of EGDT trials was unblinded. The adverse hemodynamic or sudden cardiopulmonary events that occur as a result of the transition and turnover of care from ED to ICU are diminished if care is unblinded and provided by a coordinated research or inpatient team [18].

### Antibiotic therapy

The encouragement or requisite for antibiotic administration prior to enrollment in the trio of EGDT trials is a significant intervention (Additional file 1: Table S4). An 8.5 % increase in mortality for a 6-h delay or a 7.6 % increase in mortality (septic shock) for each hour of delay from the time of diagnosis to antibiotic therapy has been observed [38, 39]. The mortality benefit of timely antibiotic administration is further enhanced by antibiotic appropriateness [38, 40].

Screening for SIRS criteria, lactate levels, fluid challenge (for hypotension), and antibiotics was a requisite for site enrollment at the centers of the trio of EGDT trials. These interventions can alter the natural trajectory of sepsis progressing to more severe disease, thus mitigating the need for aggressive intervention.

### Fluid and vasopressor therapy

From hospital arrival to the end of the 6-h study period the total fluid volume given ranged from 3.5 to 5.5 liters for the EGDT and trio of trials study groups. Overall, because of the greater lead time prior to enrollment in the trio of EGDT trials, the total volume given was actually similar to the EGDT study treatment group. The comparative differences in fluid therapy were 1482 mL (42.4 %), 697 mL (16 %), 175 mL (4.1 %), and 229 mL (4.4 %) between the EGDT and usual or control care treatment groups in the EGDT, ProCESS, ARISE, and ProMISe trials, respectively (Table 4).

In the EGDT study, the greater volume therapy or treatment effect during the 6 hours of resuscitation was associated with a greater reduction (13.8 %) in vasopressor therapy, less volume therapy (2 liters or 23 %) and lower mechanical ventilation rates (14.2 %) between the EGDT and control group during the subsequent 6- to 72-h time period.

Early and more frequent administration of vasopressors in the trio of EGDT trials may result in a hemodynamic phenotype of "vasodilatory septic shock" which is associated with a lower mortality risk as described by Hernandez et al. [41]. Waechter et al. further report that vasopressor use in the first hour may be associated with increased mortality in patients of greater illness severity [42].

### Central venous catheterization

In the EGDT trial, the timing of central venous catheter (CVC) placement was earlier compared to the trio of EGDT trials because it was an emergent standard of care provided in both treatment groups. As a result, the lower baseline CVP and ScvO_2_ values in both treatment groups are more consistent with experimental models of sepsis where hypovolemia is predominant (Table 3) [1]. The normal CVP (>10 mmHg) at study entry in the ARISE trial suggests adequate volume resuscitation at enrollment. CVC placement rates were 57.9 %, 61.9 %, and 50.9 % in the control groups of the ProCESS, ARISE, and ProMISe trials, respectively (Additional file 1: Table S2). These CVC placement rates exceed the "real life" CVC placement rate of 35.4 % noted in large observational quality improvement studies where associated mortality reduction is from 47.7 % to 29.5 % (almost identical to the EGDT study) [43]. CVC insertion (within 12 h of diagnosis) and attainment of the target CVP ≥8 mm Hg has been associated with lower in-hospital death [43, 44]. The CVC placement rates in the trio of EGDT trials potentially narrows the treatment effect between the studied groups.

### Central venous oxygen saturation (ScvO_2_)

Similar to a low CVP, a low ScvO_2_ (<40 %) is a consistent finding in experimental models and observations of early human sepsis [1, 45]. The lower ScvO_2_ values in the EGDT trial reflect earlier CVC placement, greater shock severity, or imbalances between DO_2_ (oxygen delivery) and VO_2_ (oxygen consumption) before corrective interventions [26, 46, 47]. The frequency of ScvO_2_ less than 70 % has been reported as 36 % to 45.4 % in ED patients and up to 53 % of ICU admissions. ScvO_2_ below 70 % upon ICU admission is associated with a 10.4 % increase in hospital mortality [26, 48].

Did the trio of EGDT trials shed light on the role of ScvO_2_ as an important endpoint of EGDT when the initial mean ScvO_2_ was 71 %, 72 %, and 70 % in the ProCESS, ARISE, and ProMISe trials, respectively, along with a normal CVP (Table 3)? In the EGDT trial, the mean (and median) ScvO_2_ of 49 % at randomization was 2 standard deviations below the target ScvO_2_ of 70 %. In other words, 97.5 % of enrolled patients actually required specific steps to normalize ScvO_2_. Assuming that ScvO_2_ values were normally distributed—and they were reported and analyzed in the trio of EGDT trials as parametric (normally distributed) data—the median value for ScvO_2_ would also be greater than or equal to 70 %, indicating that half of the patients had a normal baseline ScvO_2_ or reached the targeted endpoint of EGDT at the time of randomization. Assuming that randomization was effective in the trio of EGDT trials, half of patients in the usual care arms of the studies also would not have “required” specific steps of EGDT to reach this endpoint.

Intention-to-treat (ITT) analysis was a component of the trio of EGDT trials. ITT analysis is limited when the endpoint of the variable in question (ScvO_2_ and CVP) is achieved at the time of randomization [17, 49]. A methodologically more appropriate investigation would randomize patients who required normalization of ScvO_2_ (or with low baseline ScvO_2_) to receive EGDT versus usual or other forms of care. Unfortunately, without having a CVC inserted, the investigators of the trio of EGDT trials did not have a mechanism for screening those patients with low ScvO_2_ after meeting the same enrollment criteria as the EGDT trial [17].

### Myocardial dysfunction

Myocardial dysfunction can be present in up to 15 % of septic shock patients, and more frequent in the presence of cardiovascular co-morbidities [50, 51]. In addition, greater use of mechanical ventilation can potentiate adverse heart–lung interactions necessitating cardiovascular manipulation (Table 4) [52]. The hemodynamic phenotype of myocardial dysfunction (low ScvO_2_, increased CVP and lactate) may be absent on physical examination but is associated with increased mortality [53–57]. Inotropic therapy is associated with increased fluid administration as a result of reducing CVP secondary to lowering ventricular end-diastolic pressure (improving compliance) and allowing for fluid administration [58]. Ultrasound assessment has also emerged as a common tool in the ED management of shock. Recent literature suggests that left ventricular strain seen on cardiac ultrasound during sepsis is associated with a decreased ScvO_2_ and increased lactate [59]. The use of cardiac ultrasound has therefor a potential impact on therapeutic interventions used. The trio of EGDT trials did not formally discuss the use of ultrasound or other technologies (i.e., pulmonary artery catheter) and their treatment effects on usual care.

### Mechanical ventilation

The greater need for mechanical ventilation in the EGDT trial patients compared to the trio of trials is multifactorial and provides unique therapeutic and outcome dimensions (Table 4) [52, 60–62]. At enrollment, there were greater degrees of respiratory demands (increased respiratory rate and decreased partial pressure of carbon dioxide (PaCO_2_) and increasing shock severity (increased lactate and decreased ScvO_2_) in the EGDT trial patients (Table 3). Patients with acute pulmonary edema were excluded from the trio of EGDT trials without specifying a cardiogenic or non-cardiogenic etiology (acute lung injury (ALI)).

Mechanical ventilation alters the hemodynamic phenotype in severe sepsis and septic shock compared to a spontaneously breathing patient [24, 46, 60, 63]. While ScvO_2_ generally increases upon the introduction of mechanical ventilation, hemodynamic perturbations resulting from adverse heart–lung interactions may trigger more therapeutic interventions [63]. These can range from modifying the fraction of inspired oxygen, positive end-expiratory pressure, fluid administration, vasoactive agents, and decreasing the work of breathing after intubation [46, 47, 64]. Normalization of SvO_2_ even up to 47 h after disease onset is associated with improved outcomes and decreased duration of mechanical ventilation in the setting of ALI [56]. Decreased duration of mechanical ventilation is associated with more efficient and definitive shock resolution as noted up to 72 h in the EGDT group of the original trial when compared to the control group [3, 65].

The SSC database from 2005 to 2008 reports a mechanical ventilation rate of 52.4 % (7877/15,022 patients) which is almost identical to the EGDT study (Table 4). Mortality rates in this report were 48.3, 45.7, and 33.0 % in mechanically ventilated patients with ALI, without ALI, and without mechanical ventilation, respectively. In a cohort study of the Healthcare Cost and Utilization Project Nationwide Inpatient Sample, Attaway et al. also reported that mortality decreased more in sepsis patients requiring mechanical ventilation (*n* = 884,848; from 64.1 % to 39.7 %; *p* < 0.05) compared to those that did not require mechanical ventilation (*n* = 6,963,920; 14.8 % to 9.0 %; *p* < 0.05). They specifically stated that “this occurred over a decade following the introduction of EGDT (2001 to 2012)” [60]. This 24.4 % reduction in mortality is multifactorial, with protective lung strategies accounting for 8.7 % and the remainder attributed to other interventions such as EGDT [61]. The EGDT trial is unique because it was conducted before the introduction of protective lung strategies and alternative fluid management strategies [66, 67].

### Corticosteroids

Adrenal dysfunction has been found to be present in up to 19 % of vasopressor-dependent patients following adequate volume resuscitation in the ED [68]. Over 8 % of all treatment groups in the ProCESS trial received steroids prior to randomization [12]. In the ARISE trial, 36.9 % of the EGDT group versus 35.9 % of the usual care group received steroid therapy within 72 h due to co-enrollment in a double-blind randomized trial of corticosteroids in septic shock as noted in the supplemental material of the study [13]. In the ProMISe trial [14], 4–5 % and 11–12 % of both treatment groups received steroids at baseline and within 6 h, respectively (Table 4). While the impact of steroids on mortality draws continued debate, recent evidence suggests that early treatment (within 9 h) decreases the vasopressor requirement and positively impacts outcome, especially in patients with higher illness severity [69, 70]. The use of steroids for vasopressor refractory shock was absent in the EGDT trial.

### Defining usual care and other influences on mortality

The trio of EGDT trials was conducted during a period of diminishing sepsis mortality (Table 1) [5, 71, 72]. Quality improvement initiatives and other technologies implemented over this time include pre-hospital management [73], healthcare provider education [74], sepsis and antibiotic administration alerts [75], ultrasound, stroke volume, or pulse pressure variation [76–78], lactate clearance [79], scoring systems [80], rapid response teams [81], telemedicine [82], around the clock intensivist staffing [83], early referral to larger hospitals [20], palliative care [84], state-wide sepsis initiatives [16], improved coding [85], and documentation [86].

As a result of the ubiquitous nature of the SSC over the last decade, sepsis protocols or quality improvement initiatives were evident in a majority of the ProCESS trial sites before or during conduct of the trio of EGDT trials (Additional file 1: Figure S2) [87, 88]. The Sepsis Six and SEPSIS KILLS pathway were nationally implemented prior to or paralleling the ProMISe and ARISE trials and have been associated with increased resuscitation bundle compliance and improved mortality [89, 90]. These generically comprise administering high-flow oxygen, obtaining blood cultures, administering broad spectrum antibiotics and intravenous fluid challenges, and measuring serum lactate and hemoglobin along with accurate hourly urine output. The administration of supplemental oxygen can significantly increase and potentially normalizes ScvO_2_ even before enrollment [47, 64]. This is followed by reassessment and early referral to the ICU.

Compliance to the resuscitation bundle elements (lactate, cultures, antibiotics, fluid challenge, and even CVC placement) in the usual care or control groups was over 50 % in each of the trio of EGDT trials. This resulted from CVC placement as a standard of care by usual care or control care clinicians. Large observational studies have shown that even when resuscitation bundle compliance rates improve from 7 % to 29.2 %, mortality is reduced from 45.7 % to 29.5 % for an absolute risk reduction of 16.5 % and a relative risk reduction of 36 % (*p* < 0.001; Additional File 1: Figure S2) [91].

The trio of EGDT trials did not examine the impact of delayed care. A significant mortality reduction has been observed even with significant delays (up to 12 h) before initiating EGDT [92–94]. Patients could potentially receive usual or control arm care during the 6-h study period of the trio of EGDT trials and then receive delayed EGDT or a facsimile, thus altering the treatment effect between groups.

### What is the true baseline, control group or usual care mortality?

Mortality from severe sepsis and septic shock over the decade prior to and paralleling the conduct of the trio of EGDT trials has undergone a consistent and significant decrease (Table 1). For the most common cause of sepsis requiring hospital admission (pneumonia) in the USA, inpatient mortality rates have decreased 45 % among adults between 2002 and 2012 [95]. Whether protocolized care study designs were quasi experimental, prospective observational, prospective with historical controls, or retrospective or randomized controlled, an historical baseline mortality approaches 40–50 % (Table 1). These findings are consistent with a Sepsis International Consensus Definitions Task Force who performed a systematic review comprising 52 studies and 166,479 patients (1993–2015) and reported a septic shock-associated crude mortality of 46.5 % [96]. This reference mortality is identical to the original EGDT trial [3], which supports its external validity and is nearly twice the mortality of the trio of EGDT trials (Table 1) [12–14]. Using this reference mortality as usual or control group mortality, it is clear that mortality has diminished over the last decade. This significant change in mortality, if not accounted for, increases the likelihood of an underpowered trial [19].

What is the real baseline mortality in the trio of EGDT trials? When the observed hospital mortality is subtracted from the predicted mortality using the baseline mean Acute Physiology and Chronic Health Evaluation (APACHE) II scores, a relative risk mortality reduction of 45–51.5 % in ProCESS, 25.2–30.9 % in ARISE, and 24.6–25.6 % in ProMISe is seen across all treatment groups. These mortality projections similarly compare to the original EGDT trial’s relative mortality reduction of 24.3 % between treatment and control groups (Table 6).

**Table 6:** Outcomes across the EGDT, ProCESS, and ARISE trials

	EGDT		ProCESS			ARISE		ProMISe	
	EGDT	Control	EGDT	PBST	UC	EGDT	UC	EGDT	UC
APACHE II at enrollment	21.4 ± 6.9	20.4 ± 7.4	20.8 ± 8.1	20.6 ± 7.4	20.7 ± 7.5	15.4	15.8	18.7 ± 7.1	18.0 ± 7.1
Predicted mortality, % based on APACHE II	40.3	36.9	38.2	37.5	37.9	21.0	21.0	30.2	29.1
In-hospital mortality, % (actual)	30.5	46.5	21.0	18.2	18.9	14.5	15.7	25.6	24.6
Predicted minus actual mortality, %	9.8	−9.6	17.2	19.3	19.0	6.5	5.3	4.6	4.5
Relative risk reduction in hospital mortality	24.3	−26.0	45.0	51.5	50.1	30.9	25.2	25.6	24.6
Incidence of cardiovascular complications %	10	20	5.2	4.9	8.1	7.1	5.3	2.1	1.6

*APACHE II* Acute Physiology and Chronic Health Evaluation II, *ARISE* Australasian Resuscitation in Sepsis Evaluation, *EGDT* Early Goal-Directed Therapy, *PBST* protocol-based standard therapy, *ProCESS* Protocolized Care for Early Septic Shock, *ProMISe* Protocolized Management of Sepsis, *UC* usual care

### Hemodynamic subgroups (phenotypes)

Early risk stratification of high-risk patients using SIRS, lactate screening, and fluid challenge was a standard of care in all treatment groups in the trio of EGDT trials. Lactate screening not only provides earlier detection of occult shock, but therapeutically alters the natural history of sepsis by decreasing early cardiopulmonary events, which can occur in up to 20 % of patients [3, 97, 98]. These events associated with increased mortality can range from hypotension, respiratory deterioration, and cardiac arrhythmias, to cardiac arrest even after the 6-h trial period and ICU admission [98, 99]. Early lactate screening further leads to a reduction in the time to overall diagnostic results, intravenous fluids, ED care, and ICU admission. The estimated mortality reduction attributed to lactate screening approaches 11 % [100, 101].

In the EGDT trial, the baseline lactate level was almost 2 mM/L higher than in the trio of EGDT trials. The number of patients with a lactate less than 4 mM/L was 21 %, 45 %, 54 %, and 35.4 % in the EGDT, ProCESS, ARISE, and ProMISe trials, respectively (Table 3) [3, 12–14]. In the ProCESS trial, the number of patients with a lactate greater than 5.3 mM/L was 12 % higher in the EGDT group compared to the two other study groups (*p* = 0.05).

**Fig. 2:**
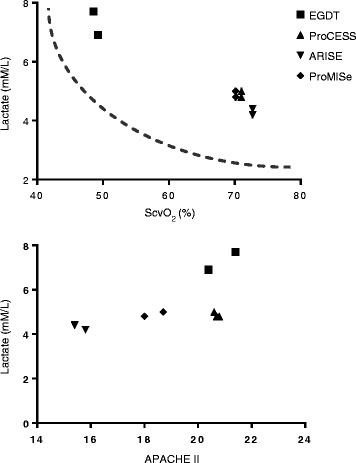
Comparing baseline lactate to ScvO_2_ and APACHE II scores. *APACHE II* Acute Physiology and Chronic Health Evaluation II, *ARISE* Australasian Resuscitation in Sepsis Evaluation, *EGDT* Early Goal-Directed Therapy, *ProCESS* Protocolized Care for Early Septic Shock, *ProMISe* Protocolized Management in Sepsis, *ScvO*_*2*_ mixed central venous oxygen saturation

The combination of a high lactate and low ScvO_2_ at baseline comprise a hemodynamic phenotype that is independently associated with greater degrees of systemic inflammation, organ dysfunction, and higher mortality [31, 97, 100, 102–105] (Fig. 2). When this hemodynamic phenotype is adjusted for organ dysfunction (lactate/APACHE II ratio) at baseline, patients in the EGDT trial are also of higher acute illness severity compared to the trio of EGDT trials (Fig. 2) [26, 27, 48, 106].

## Conclusions

EGDT has been shown to have internal and external validity in reducing mortality for the treatment of severe sepsis and septic shock. The various approaches examined by the trio of EGDT trials suggest that alternative strategies can provide an equal reduction in mortality. However, as a result of multiple methodological differences when compared to the original EGDT trial (including undefined usual care), the external validity of these alternative strategies remain to be determined. The combination of a diminishing treatment effect between these alternative strategies and EGDT, along with a global reduction in sepsis mortality over the last 15 years, can render even well-conducted control trials underpowered and inconclusive.

The trio of EGDT trials provides enormous insight into explaining the discrepancy in trials attempting to replicate a previously positive trial over a decade later. It has been shown that large prospective observational studies which have confirmed the external validity and reliability of the EGDT trial provide an equally reliable scientific alternative to randomized control trials [17–19, 107, 108]. 

In this era of global reductions in sepsis mortality, clinicians should view EGDT as a verb (series of actions) rather than a noun. Future research should focus on the precision for using invasive or non-invasive approaches at the initial presentation of high risk patients.

## Additional file

Additional file 1: Online Supplemental Information. **Figure S1**. Oxygen transport and utilization. **Figure S2**. Changes in mortalities over time in regard to before and after Implementation of EGDT. **Figure S3**. Pre and Post-Randomization Study Workflow Comparisons. **Table S1**. Enrollment characteristics and data. T**able S2**. Comparison of enrollment criteria and resuscitation end-points. **Table S3**. Comparison of treatments across the EGDT, ProCESS, ARISE, and ProMISe trials. **Table S4**. Patient enrollment and treatment initiation. (DOC 543 kb)

## Abbreviations

ALI: acute lung injury
APACHE: Acute Physiology and Chronic Health Evaluation
ARISE: Australasian Resuscitation in *S*epsis Evaluation
CVC: central venous catheter
CVP: central venous pressure
ED: emergency department
EGDT: Early Goal-Directed Therapy
ICU: intensive care unit
ITT: intention-to-treat
ProCESS: Protocol-Based Care for Early Septic Shock
ProMISe: Protocolized Management in Sepsis
ScvO_2_: central venous oxygen saturation
SIRS: systemic inflammatory response syndrome
SSC: Surviving Sepsis Campaign
SvO_2_: mixed venous oxygen saturation
