# Supplementary material for: Early goal-directed therapy in severe sepsis and septic shock: insights and comparisons to ProCESS, ProMISe, and ARISE
Source: Crit Care. 2016 Jul 1;20:160. doi: 10.1186/s13054-016-1288-3 (PMC4929762; doi:10.1186/s13054-016-1288-3)
Supplement: Additional file 1: — Online Supplemental Information. Figure S1. Oxygen transport and utilization. Figure S2. Changes in mortalities over time in regard to before and after Implementation of EGDT. Figure S3. Pre and Post-Randomization Study Workflow Comparisons. Table S1. Enrollment characteristics and data. Table S2. Comparison of enrollment criteria and resuscitation end-points. Table S3. Comparison of treatments across the EGDT, ProCESS, ARISE, and ProMISe trials. Table S4. Patient enrollment and treatment initiation. (DOC 543 kb) [file 13054_2016_1288_MOESM1_ESM.doc]

**Online Supplement**

**Figure Legend**

Figure S1. Oxygen transport and utilization

Figure S2. Changes in mortalities over time in regard to before and after implementation of EGDT

Figure S3. Pre and Post-Randomization Study Workflow Comparisons

**Figure S1.** Oxygen delivery and consumption. As shock progresses toward DO_2 crit_, oxygen extraction ratio (OER) increases, resulting in decreased ScvO_2_/SvO_2_. Lactate increases as a result of global tissue hypoxia, signifying oxygen debt.


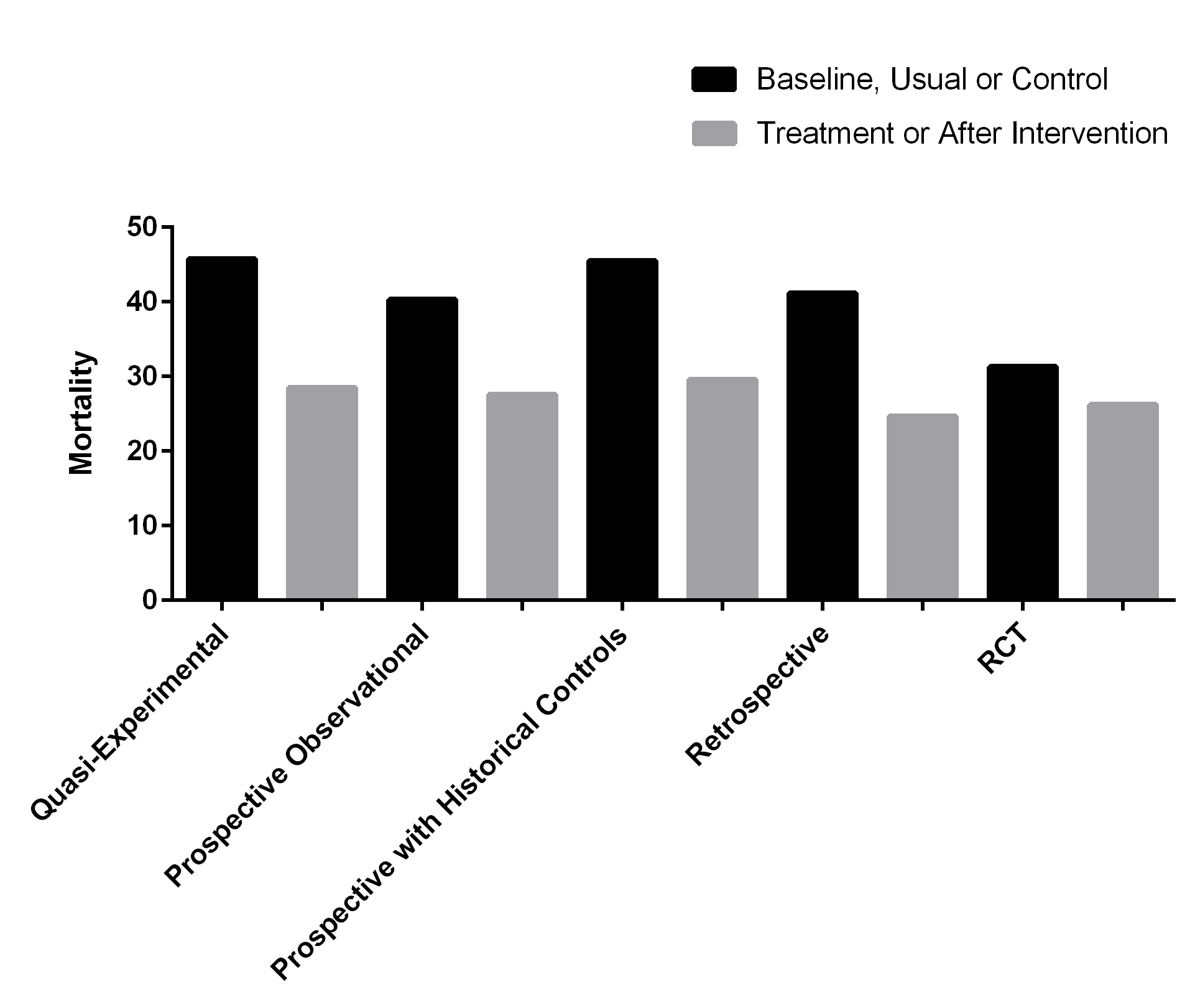


Figure S2. Changes in mortalities over time in regard to before and after implementation of EGDT

| **Studies of EGDT** | **Number of Studies** | **Number of Patients** | **Mortality (%)**  **(Before, Baseline,**  **Usual or Control)** | **Mortality (%)**  **(After, Treatment)** |
| --- | --- | --- | --- | --- |
| Quasi experimental studies [137-140]  Prospective Observational [43, 87, 91, 93,141-173]  Prospective with historical controls [175-183]  Retrospective [184-193]  Randomized Control Trials [3, 12-14, 79, 194-199] | 4  38  9  10  11 | 1120  66862  2250  2183  5756 | 45.8  40.3  45.5  41.1  31.3 | 28.5  27.6  29.6  24.7  26.2 |


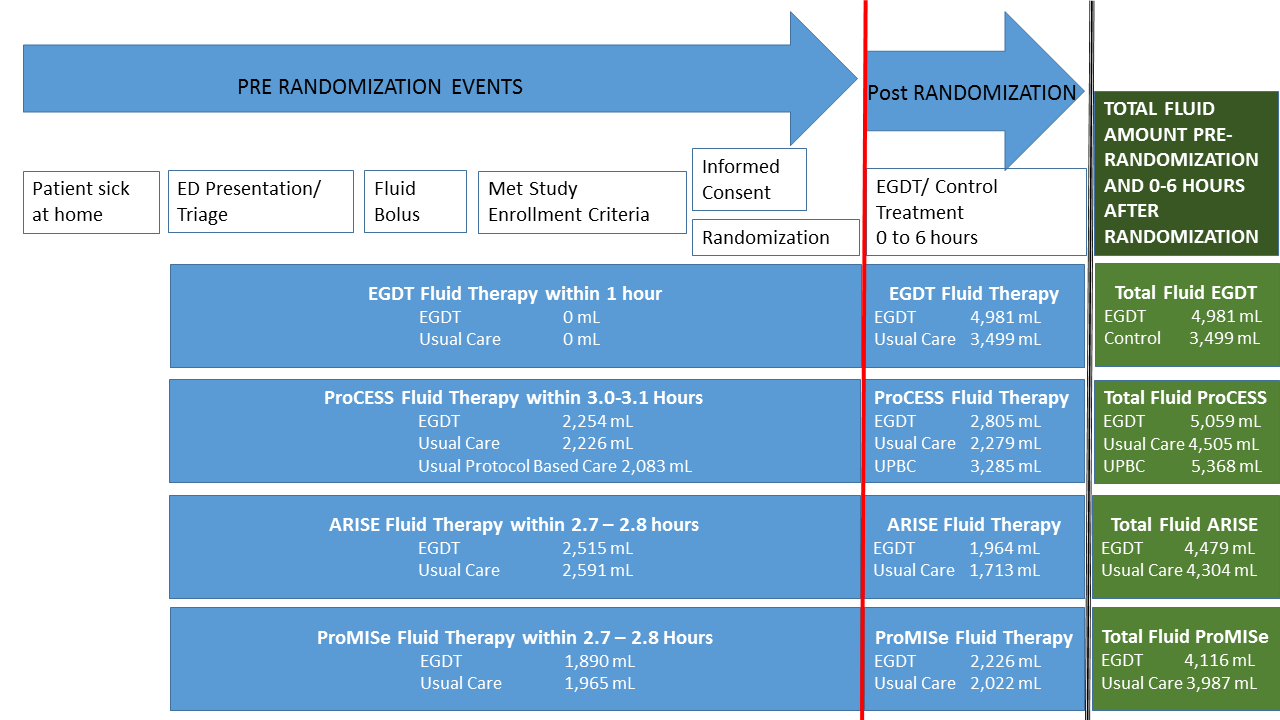


Figure S3. Pre and Post-Randomization Study Workflow Comparisons

ARISE, Australasian Resuscitation in Sepsis Evaluation; ED, emergency department; EGDT Early Goal-Directed Therapy; ProCESS, Protocolized Care for Early Septic Shock; ProMISe, Protocolized Management in Sepsis; UPBC, Usual Protocol Based Care

**Table S1. Enrollment characteristics and data**

|  | **EGDT** | **ProCESS** | **ARISE** | **ProMISe** |
| --- | --- | --- | --- | --- |
| Location | United States | United States | Multinational^*^ | United Kingdom |
| Number of centers | 1 | 31 | 51^*^ | 56 |
| Setting | Metropolitan academic teaching hospital | Metropolitan academic teaching hospitals | Metropolitan and rural tertiary and non-tertiary care teaching hospitals | National Health Service hospitals throughout the United Kingdom |
| Hospital size  (number of beds) | 850 | Not reported | 120-930  (up to 1800 in  Hong Kong) | 234-1313 |
| Average yearly ED visits | 90,000 | > 40,000 | 15,000-90,000 (up to 200,000 in Hong Kong) | 46,761-185,000 |
| Enrollment time frame | March 1997-March 2000 | March 2008-May 2013 | October 2008-April 2014 | February 2011-July 2014 |
| Duration of study (months) | 36 | 62 | 66 | 41 |
| Patients enrolled | 263 | 1341 | 1600 | 1260 |
| Eligible patients excluded | 10.4% | 65.0 % | 42.7% | 66.6% |
| Enrollment/month/center | 7 | 0.7 | 0.5 | 0.5 |
| Patients per study arm |  |  |  |  |
| EGDT | 130 | 439 | 796 | 630 |
| Control | 133 |  |  |  |
| Usual Care |  | 456 | 804 | 630 |
| PBST |  | 446 |  |  |
| Lactate screening | None | Required | Required | Required |
| Existing sepsis protocols  (SSC-2004, 2008, 2012) | No | Yes (SSC and individual centers) | Yes (SSC and national standards) | Yes (SSC and national standards) |

^*^Number of study sites by Country Australia (42), New Zealand (3), Finland (2), Ireland (1) and Hong Kong (3). ARISE, Australasian Resuscitation in Sepsis Evaluation; ED, emergency department; EGDT, early goal-directed therapy; PBST, protocol-based standard therapy; ProCESS, Protocolized Care for Early Septic Shock; ProMISe, Protocolized Management in Sepsis; SSC, Surviving Sepsis Campaign.

**Table S2. Comparison of enrollment criteria and resuscitation end-points**

|  | **EGDT** | | **ProCESS** | | | | **ARISE** | | **ProMISe** | |
| --- | --- | --- | --- | --- | --- | --- | --- | --- | --- | --- |
|  | EGDT | Control | EGDT | | PBST | UC | EGDT | Control | EGDT | Control |
| Temperature, °C | 35.9 | 36.6 | 37.6 | | 37.6 | 37.7 | 37.6 | 37.6 |  |  |
| Heart rate, beats/min | 117 | 114 | 113.7 | | 114.6 | 114.5 | 104.9 | 104.7 |  |  |
| Systolic blood pressure, mm Hg | 106 | 109 | 100.2 | | 102.1 | 99.9 | 78.8 | 79.6 | 77.7 | 78.4 |
| Respiratory rate, breath/min | 31.8 | 30.2 | 25.4 | | 25.1 | 25.3 | 24.5 | 25.1 |  |  |
| MAP, mm Hg | 74 | 76 | 65 | | 66 | 65 | 69.4 | 70.5 | 58.8 | 59.0 |
| Lactate, mM/L | 7.7 | 6.9 | 4.8 | | 5.0 | 4.8 | 4.4 | 4.2 |  | 5.1 |
| Lactate before enrollment, mM/L |  |  |  | |  |  | 6.7 | 6.6 | 7.0 | 6.8 |
| Lactate < 4, mM/L (%) | 21 | | 45 | | | | 54 | | 35.4 | |
| Lactate > 4, mM/L (%) | 79 | | 59 | | 59.2 | 60.7 | 46 | 46.5 | 65.4 | 63.7 |
| CVC catheterization % | All | All | 93.6 | | 56.5 | 57.9 | 90 | 61.9 | 92.1 | 50.9 |
| CVP, mmHg (Initial) | 5.3 | 6.1 |  | |  |  | >10 |  |  |  |
| pH | 7.31 | 7.32 | 7.33 | | 7.31 | 7.34 |  |  |  |  |
| PaCO_2_, mm Hg | 31.5 | 30.6 | 35.7 | | 38.9 | 36.9 | 35.2 | 35.5 |  |  |
| White blood count, per mm^3^ | 13,600 | 14,200 | 15,300 | | 15,600 | 16,800 | 13,500 | 13,700 |  |  |
| Creatinine, mg/dL | 2.6 | 2.6 | 2.5 | | 2.2 | 2.3 | 1.43 | 1.5 |  |  |
| Hematocrit, % | 34.5 | | 35.1^*^ | | | | 37.8 | |  |  |
| Blood urea nitrogen, mg/dL | 47.1 | 45.4 | 35.1 | 32.5 | | 35.6 |  |  |  |  |
| Total bilirubin, mg/dL | 1.3 | 1.9 |  |  | |  | 0.93 | 0.99 |  |  |
| APACHE II Score | 21.4 | 20.4 | 20.7 | 20.6 | | 20.8 | 15.4 | 15.8 | 18.7 | 18.0 |
| Lactate (6 hours) mM/L | 4.3 | 4.9 |  |  | |  | 2.8 | 2.9 |  |  |
| Lactate clearance (6 hours, %) | 44 | 29 |  |  | |  | 58.2 | 56.1 |  |  |
| MAP (6 hours), mm Hg | 95 | 81 | 77 | 79 | | 76 | 76.5 | 75.3 | 76.5 | 76.5 |
| MAP > 65 mm Hg (6 hours) | 100 | 100 | 83.1 | 84.1 | | 77.2 | 94.1 |  |  |  |
| CVP, mmHg (6 hours) | 13.8 | 11.8 |  |  | |  | 11.4 | 11.9 | 11.2 | 11.7 |
| ScvO_2_, % | 48.6 | 49.2 | 71 | 72.7 | |  | 70.12 |  |  |  |
| ScvO_2_ (initial, %) | 48.6 | 49.2 | 71 |  | |  | 72.7 |  |  |  |
| ScvO_2_ (6 hours, %) | 77.3 | 66.0 |  |  | |  | 75.9 |  | 74.2 |  |
| ScvO_2_ > 70% (6 hours, %) | 94.9 | 60.2 |  |  | |  | 95.3 |  |  |  |

APACHE II, Acute Physiology and Chronic Health Evaluation II; ARISE, Australasian Resuscitation in Sepsis Evaluation; CVC, central venous catheterization; CVP, central venous pressure; EGDT, early goal-directed therapy; MAP, Mean Arterial Pressure; MODS, Multiple Organ Dysfunction Score; NR, not reported; PaCO_2_, partial pressure of carbon dioxide; ProCESS, Protocolized Care for Early Septic Shock; ProMISe, Protocolized Management in Sepsis; ScvO_2_, central venous oxygen saturation; WBC, white blood cells. ^*^ProCESS reported hemoglobin only; Conversion hematocrit = hemoglobin x 3.

**Table S3. Comparison of treatments across the EGDT, ProCESS, ARISE, and ProMISe trials.**

|  | **EGDT** | | **ProCESS** | | | **ARISE** | | **ProMISe** | |
| --- | --- | --- | --- | --- | --- | --- | --- | --- | --- |
|  | EGDT | Control | EGDT | PBST | UC | EGDT | UC | EGDT | UC |
| Fluids prior to enrollment, mL |  |  | 2,254 | 2,226 | 2,083 | 2,515 | 2,591 | 1950 | 2,000 |
| Fluid per prior to enrollment, mL/kg |  |  | 30.5 | 29.2 | 28.0 | 34.6 | 34.7 |  |  |
| Fluids 0-6 hours, mL | 4,981 | 3,499 | 2,805 | 3,285 | 2,279 | 1,964 | 1,713 | 2,226 | 2,022 |
| Fluids 6-72 hours, mL | 8,625 | 10,602 | 4,428 | 4,896 | 4,354 | 4,274 | 4,382 | 4,215 | 4,366 |
| Total fluids 0-72 hours mL | 13,443 | 13,358 | 7,720 | 8,175 | 6,663 | 6,906 | 6,672 | 5,946 | 5,844 |
| Colloid 0-6 hours, mL* |  |  |  |  |  | 323 | 249 | 1,000 | 750 |
| Colloid 6-72 hours, mL* |  |  |  |  |  | 345 | 328 | 750 | 750 |
| Vasopressor at enrollment, % |  |  | 19.1 | 16.8 | 15.1 | 21.0 |  | 2.4 | 3.4 |
| Vasopressor 0-6 hours, % | 27.4 | 30.3 | 54.9 | 52.2 | 44.1 | 66.6 | 57.8 | 53.3 | 46.6 |
| Vasopressor 6-72 hours, % | 29.1 | 42.9 | 19.8 | 20.9 | 18.0 | 58.8 | 51.5 | 57.9 | 52.6 |
| Vasopressor 0-72 hours, % | 36.8 | 51.3 | 27.3 | 24.0 | 22.4 |  |  | 60.5 | 55.0 |
| Inotrope enrollment, % | 0 | 0 | 0 | 0 | 0 |  |  | 0.3 | 0.0 |
| Inotrope 0-6 hours, % | 13.7 | 0.8 | 8.0 | 1.1 | 0.9 | 15.4 | 2.6 | 18.1 | 3.8 |
| Inotrope 6-72 hours, % | 14.5 | 8.4 | 4.3 | 2.0 | 2.2 | 9.5 | 5.0 | 17.7 | 6.5 |
| Any inotrope, % | 15.4 | 9.2 | 9.3 | 2.5 | 2.9 |  |  |  |  |
| Mechanical ventilation 0-6 hours, % | 53.0 | 53.8 | 26.4 | 24.7 | 21.7 |  |  | 20.2 | 19.0 |
| Mechanical ventilation 6-72 hours, % | 2.6 | 16.8 | 33.7 | 31.4 | 27.9 |  |  | 24.4 | 25.4 |
| Any mechanical ventilation, % | 55.6 | 70.6 | 36.2 | 34.1 | 29.6 | 30.0 | 31.5 | 27.4 | 28.5 |
| Duration of respiratory organ support, hours (days) | 9.0 | 9.0 | 6.4 | 7.7 | 6.9 | 2.6 | 2.8 | 19.6^*^ | 19.8^*^ |
| PRBC transfusion at enrollment, % |  |  | 1.1 | 1.6 | 2 |  |  | 4 | 10 |
| PRBC 0-6 hours, % | 64.1 | 18.5 | 14.4 | 8.3 | 7.5 | 13.6 | 7.0 | 8.8 | 3.8 |
| PRBC 6-72 hours, % | 11.1 | 32.8 | 4.3 | 2.0 | 2.2 | 11.0 | 11.8 | 12.6 | 8.5 |
| Any PRBC, % | 68.4 | 44.5 | 9.3 | 2.5 | 2.9 |  |  |  |  |
| Steroids at enrollment, % | None | None | 9.3 | 9.4 | 8.3 |  |  | 5 | 4 |
| Steroids 6 hours, % | None | None | 12.3 | 10.8 | 8.1 |  |  | 11.7 | 11.5 |
| Any steroids 72 hours, % | None | None |  |  |  | 36.9 | 35.9 | 21.9 | 21.1 |

ARISE, Australasian Resuscitation in Sepsis Evaluation; EGDT, early goal-directed therapy; PBST, protocol-based standard therapy; PRBC, packed red blood cells; ProCESS, Protocolized Care for Early Septic Shock; ProMISe, Protocolized Management in Sepsis; hrAPC, human recombinant activated protein C; UC, usual care. ^*^The volume effect of administered colloid solution depends on the dosage strength of the colloid. A 5% albumin solution has a volume expansion effect of 70%-100%, a 20%-25 % albumin solution as a volume expansion effect of 300%-500%.

**Table S4. Patient enrollment and treatment initiation**

|  | **EGDT** | | **ProCESS** | | | **ARISE** | | ProMISe | |
| --- | --- | --- | --- | --- | --- | --- | --- | --- | --- |
|  | EGDT | Control | EGDT | PBST | Usual | EGDT | Control | EGDT | Usual |
| Location of screening | ED | | ED | | | ED | | ED | |
| Fluid challenge | 20-30 mL/kg | | Initially, 20 mL/kg; changed to 1000 mL (55% enrolled using latter criteria) | | | 1000 mL (70%) | | 1000 mL | |
| Time from ED arrival to randomization (hours) | 1.3 | 1.5 | 3.3 | 3.1 | 3.0 | 2.8 | 2.7 | 2.5 | 2.5 |
| Antibiotics | Within 6 hours | | Before enrollment | | | Before enrollment | | Before enrollment | |
| Duration to first dose of antibiotics (minutes) | Not reported | | Not reported | | | 70 | 67 | Not reported | |
| ED length of stay (hours) | 8.0 | 6.3 | Not reported | | | 1.4 | 2.0 | 1.2 | 1.2 |
| Location of treatment initiation | ED | | ED | | | ED/ICU | | ED/ICU | |
| Blinding to treatment allocation | Yes | | No | | | No | | No | |
| Treatment team structure | ED attending, 2 residents, 3 nurses | | Study physician/attending, study coordinator, nurse | | | ED or ICU MD consultant, registrar,  or nurse | | ED or ICU MD consultant, registrar,  or nurse | |

ARISE, Australasian Resuscitation in Sepsis Evaluation; ED, emergency department; EGDT, early goal-directed therapy; ICU, intensive care unit; PBST, protocol-based standard therapy; ProCESS, Protocolized Care for Early Septic Shock; ProMISe, Protocolized Management in Sepsis
